# Expression of Alanine-Serine-Cysteine Transporter 2 (ASCT2) and L-type Amino Acid Transporter 1 (LAT1) in Low-Grade and High-Grade Gliomas: An Analysis of Open-Access Immunohistochemical Data

**DOI:** 10.7759/cureus.88325

**Published:** 2025-07-19

**Authors:** William T Han, Nina Cheng, Najib Muhammad, John Y.K. Lee

**Affiliations:** 1 Department of Neurosurgery, University of Pennsylvania Perelman School of Medicine, Philadelphia, USA

**Keywords:** amino acid transporters, asct2, diagnostic and theranostic targets, glioma prognosis, lat1, low-grade glioma

## Abstract

Background: The amino acid transporters alanine-serine-cysteine transporter 2 (ASCT2) and L-type amino acid transporter 1 (LAT1) are the primary transporters of essential amino acids in cancer. ASCT2 and LAT1 are overexpressed in solid tumors and have been associated with poor survival. These transporters may hold prognostic significance and offer potential theranostic utility in the treatment of glioma. The aims of this study are twofold: (1) to determine whether ASCT2 and LAT1 mRNA expression are related to glioma grade and survival; and (2) to characterize ASCT2 and LAT1 expression in low-grade glioma (LGG) and high-grade glioma (HGG).

Methods: Survival and mRNA expression data for both ASCT2 and LAT1 were obtained from The Cancer Genome Atlas (TCGA), accessed via the cBio Cancer Genomics Portal. GraphPad Prism was used to conduct a Kaplan-Meier survival analysis. Immunohistochemistry images were obtained from the Human Protein Atlas (HPA) database and analyzed with QuPath for ASCT2 and LAT1 expression in the cytoplasm and at the endothelium.

Results: Query of TCGA resulted in 49 samples from 28 patients, which were evaluated for ASCT2 and LAT1 expression. Cytoplasmic staining of mRNA for ASCT2 and LAT1 was not associated with glioma grade or survival. Higher mRNA expression of ASCT2 correlated with worse survival (p=0.002) in LGG but not in HGG. mRNA expression of LAT1 did not have any prognostic value. QuPath analysis of samples from the HPA showed increased staining for both ASCT2 and LAT1 at the endothelium in both LGG and HGG. Endothelial expression of ASCT2 and LAT1 correlated with glioma grade.

Conclusion: In our small study conducted with open-access data, ASCT2 and LAT1 did not appear to have prognostic value. However, both ASCT2 and LAT1 staining were increased at the endothelium compared to normal brain tissue. These amino acid transporters may play an important role in tumor proliferation and support theranostic approaches to glioma.

## Introduction

Malignant tumors have an increased demand for protein synthesis in order to meet the needs of rapidly dividing cells. Transporters play a central role in the uptake of amino acids necessary for cancer cell growth, maintaining cellular nitrogen levels, cellular signaling, and growth regulation [1-3]. As such, amino acid transporters represent a potential cancer prognostic marker and therapeutic target. 

Two amino acid transporters involved in neuronal regulation have been implicated in glioma development and proliferation: the neutral amino acid transporters solute carrier family A1 member 5/alanine-serine-cysteine transporter 2 (ASCT2) and solute carrier family A7 member 5/L-type amino acid transporter 1 (LAT1) [3,4]. ASCT2, which exists as a homotrimer, is a Na⁺-dependent antiporter with an affinity for neutral amino acids, including glutamine, which is the principal precursor for glutamate and GABA [2,5,6]. LAT1, which exists as a functional heterodimer with a glycoprotein heavy chain named 4F2hc, is a Na⁺-independent antiporter that transports bulky hydrophobic amino acids [2,7]. LAT1 is expressed 100-fold higher at the blood-brain barrier (BBB) than in other tissues, and has been shown to transport multiple drugs across the BBB [8].

Previous groups have demonstrated increased expression of amino acid transporters, including ASCT2 and LAT1, in metastatic brain cancer as well as in cancers of the kidney, colon, endometrium, stomach, liver, lung, ovary, pancreas, and prostate [2,9]. Both transporters are thought to contribute to cancer pathogenesis in a variety of ways, such as through increased resistance to cell death and mTOR proliferative signaling [8]. In gliomas, LAT1 has been associated with angiogenesis [10]. Increased expression of ASCT2, in particular, has been implicated in areas where glutamine plays an important metabolic role, such as the liver, gut epithelium, and brain [2].

High LAT1 and ASCT2 gene expression levels have been associated with poor survival [9,11,12]. Compared to other cancers, the prognostic value of LAT1 and ASCT2 expression in gliomas has not been studied extensively. LAT1 expression has been associated with glioma phenotype, with higher staining observed in high-grade gliomas (HGG) than in low-grade gliomas (LGG) in case series from two centers in Japan [10,13]. LAT1 hyperexpression was associated with decreased overall survival among glioblastomas in a small case series from Indonesia [14]. Studies of ASCT2 expression in glioma have not been previously published.

Hyperexpression of LAT1 and ASCT2 may be useful for differentiating LGG from HGG, which would be expected to have prognostic significance. This study seeks to augment current understanding regarding ASCT2 and LAT1 expression in LGG and HGG and their prognostic implications, utilizing a public database (1) to evaluate the association between LAT1 and ASCT2 hyperexpression and survival among LGG and HGG, and (2) to characterize ASCT2 and LAT1 expression in LGG and HGG.

## Materials and methods

mRNA expression versus survival in HGG and LGG for ASCT2 and LAT1

The Cancer Genome Atlas (TCGA) (http://cancergenome.nih.gov/) provides publicly available datasets accessed via the cBio Cancer Genomics Portal (https://www.cbioportal.org/) (cBio) for the exploration of cancer genomics [15]. Two datasets published by TCGA’s Pan-Cancer Atlas, Brain Lower Grade Glioma and Glioblastoma Multiforme, were queried for patients with previously untreated LGG or HGG, respectively, for whom mRNA expression and survival data were available (PMID: 29625048) [15]. The classification into LGG and HGG was assigned by TCGA. mRNA expression data, baseline patient characteristics, and survival data were extracted from the database.

Log-transformed mRNA expression z-scores from both datasets were downloaded from cBio. mRNA expression z-scores for ASCT2 and LAT1 were then categorized into high-expression or low-expression groups, defined as expression levels above the 75th percentile or below the 25th percentile, respectively.

Kaplan-Meier functions and log-rank tests were used to compare survival between high- and low-expression groups. Differences between groups were considered significant at a p-value of <0.05. All statistical analyses were performed using GraphPad Prism 5.0 (GraphPad Software, Inc., San Diego, CA).

Immunohistochemistry staining, image analysis, and tumor grade method

The Human Protein Atlas (HPA) (http://proteinatlas.org/) is an open-access resource and currently serves as the largest database dedicated to mapping the spatial distribution of human proteins in tissues, cancers, and cell lines (PMID: 18853439) [16]. The HPA was queried for normal and glioma brain samples.

Images of IHC slides stained with anti-LAT1 antibody HPA052673 and anti-ASCT2 antibody HPA035239, along with baseline patient characteristics, were downloaded. The IHC slides were processed by the HPA using a DAB-based chromogenic staining protocol with hematoxylin counterstain, as described on their website. Initial analysis of antibody staining was performed by the HPA; staining levels were categorized by the fraction of stained cells (<25%, 25-75%, or >75%).

We additionally quantified antibody staining of the endothelium in all three groups: LGG, HGG, and normal brain. Using the QuPath positive cell detection tool, a region of interest was drawn around the endothelium by a blinded observer without prior knowledge of the pathological diagnosis. The percentage of stained endothelial cells within this region was compared to the percentage of stained cells in the tissue as a whole.

## Results

For survival analysis, the query of TCGA resulted in a total of 251 LGG patients and 74 HGG patients with expression data for LAT1 and ASCT2 (Table 1). Patients were divided into high-expression and low-expression groups for LAT1 and ASCT2. Low expression of LAT1 was found in 124 patients with LGG and 35 patients with HGG. High expression of LAT1 was found in 127 patients with LGG and 39 patients with HGG. Low expression of ASCT2 was found in 123 patients with LGG and 37 patients with HGG. High expression of ASCT2 was found in 128 patients with LGG and 37 patients with HGG.

**Table 1:** Baseline characteristics Query of TCGA resulted in a total of 251 LGG patients and 74 HGG patients. Patients were divided into high-expression and low-expression groups for LAT1 and ASCT2, as shown above. ASCT2, alanine-serine-cysteine transporter 2; LAT1, L-type amino acid transporter 1; LGG, low-grade glioma; HGG, high-grade glioma; TCGA, The Cancer Genome Atlas

		Low grade	High grade
Total patients		251	74
Females	LAT1	118	20
	ASCT2	114	24
Males	LAT1	136	31
	ASCT2	138	34
Mean diagnosis age	LAT1	43.2 (SD=13.5)	62.44 (SD=13.58)
	ASCT2	43.7 (SD=13.6)	62.09 (SD=13.75)
LAT1	Low expression	124	35
	High expression	127	39
ASCT2	Low expression	123	37
	High expression	128	37

**Figure 1:**
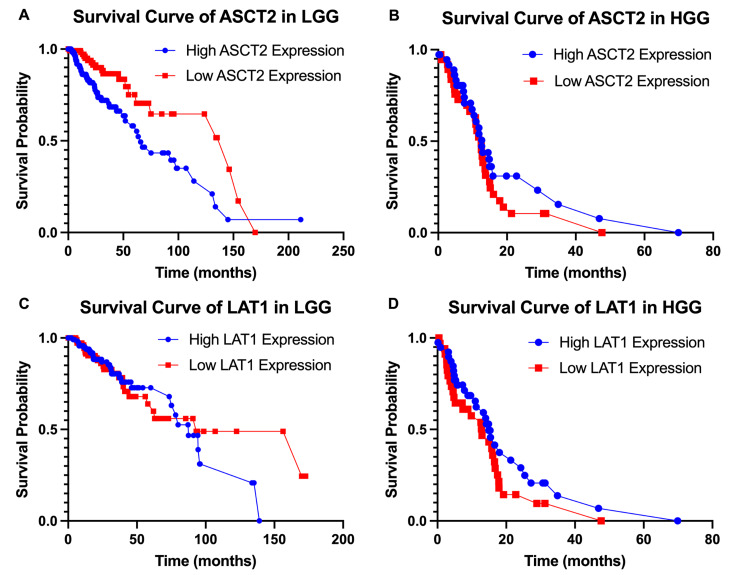
Expression versus survival comparison for ASCT2 and LAT1 For LGG, high ASCT2 mRNA expression was associated with decreased overall survival compared to the low expression group (p-value=0.002) (A). Overall survival was similar between LAT1 mRNA expression groups (p-value=0.359) (C). For HGG, no significant differences in overall survival were observed between high and low LAT1 (p-value=0.289) or ASCT2 (p-value=0.176) mRNA expression groups (B, D). ASCT2, alanine-serine-cysteine transporter 2; LAT1, L-type amino acid transporter 1; LGG, low-grade glioma; HGG, high-grade glioma

For LGG, high ASCT2 mRNA expression was associated with decreased overall survival compared to the low-expression group (p=0.002) (Figure 1A). Overall survival was similar between LAT1 mRNA expression groups (p=0.359) (Figure 1C). For HGG, no significant differences in overall survival were observed between high and low LAT1 (p=0.289) or ASCT2 (p=0.176) mRNA expression groups (Figures 1B, 1D).

For qualitative analysis, the HPA query yielded 28 patients (49 samples) with anti-ASCT2 or anti-LAT1 staining of gliomas or normal brain tissue (Table 2). Fifteen patients (26 samples) were included in the ASCT2 expression group, and 13 patients (23 samples) in the LAT1 expression group.

**Table 2:** Immunohistochemical staining of ASCT2 and LAT1 in HGG and LGG The expression of ASCT2 or LAT1 was analyzed in 28 patients (49 samples). ASCT2 expression was assessed in samples from 15 patients: 8 HGG, 4 LGG, and 3 normal cerebral cortex. LAT1 expression was assessed in samples from 13 patients: 7 HGG, 5 LGG, and 3 normal cerebral cortex. ASCT2, alanine-serine-cysteine transporter 2; LAT1, L-type amino acid transporter 1; LGG, low-grade glioma; HGG, high-grade glioma

	Characteristics	ASCT2	LAT1	Total
Total samples	Patients	15	13	28
	Female sex	7	4	11
	Samples	26	23	49
	Mean age in years (SD)	50.6 (15.3)	49.4 (15.6)	49.0 (14.6)
HGG	Patients	8	7	15
	Samples	15	13	28
	No cytoplasmic staining (patients)	1	3	4
	<25%	5	3	8
	25-75%	0	1	1
	>75%	2	0	2
LGG	Patients	4	3	7
	Samples	8	5	12
	No cytoplasmic staining (patients)	3	2	5
	<25%	1	1	2
	25-75%	0	0	0
	>75%	0	0	0
Normal cerebral cortex	Patients	3	3	6
	Samples	3	3	6
	No cytoplasmic staining (patients)	0	0	0
	<25%	3	0	3
	25-75%	0	3	0
	>75%	0	0	0

Of the 15 patients with samples stained for ASCT2 expression, eight patients (15 samples) had HGG, four patients (eight samples) had LGG, and three patients had normal brain tissue. For HGG, one patient showed no ASCT2 expression, five patients had <25% staining, and two patients had >75% staining. For LGG, one patient showed no cytoplasmic staining, two samples had <25% staining, and no patients had 25-75% or >75% staining. For normal cerebral tissue, all three patients showed <25% ASCT2 expression.

Of the 13 patients with samples stained for LAT1 expression, seven patients (13 samples) had HGG, three patients (five samples) had LGG, and three patients had normal brain tissue. For HGG, three patients showed no LAT1 expression, three had <25% staining, one had 25-75% staining, and none had >75% staining. For LGG, two patients showed no cytoplasmic staining, and one had <25% staining. No patients with LGG showed 25-75% or >75% staining. For normal cerebral tissue, all three patients showed 25-75% LAT1 expression.

**Figure 2:**
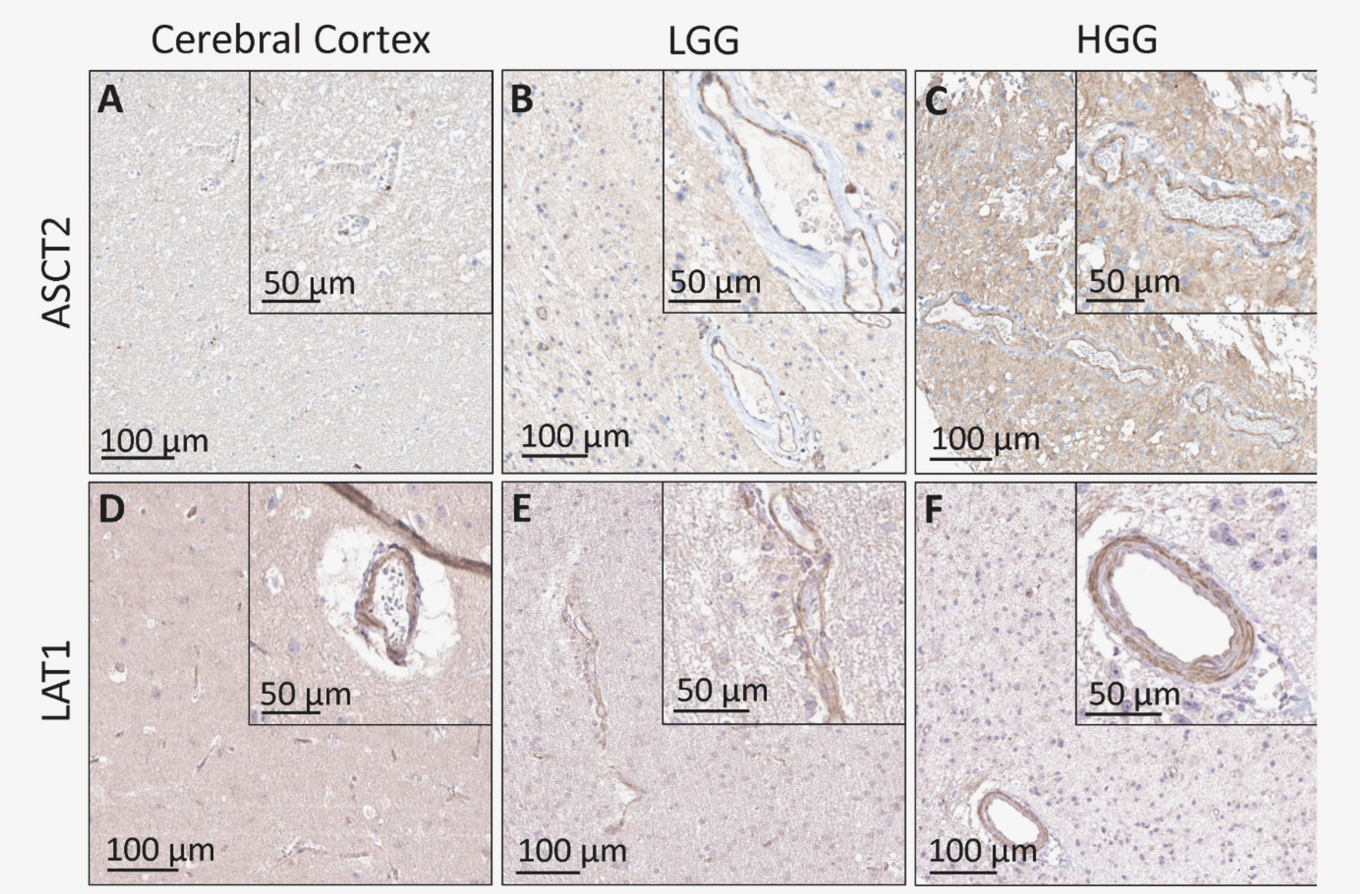
ASCT2 and LAT1 staining in normal cerebral cortex, LGG, and HGG QuPath analysis revealed that ASCT2 was expressed in (A) normal tissue at 2.05%, (B) LGG tissue at 1.22%, and (C) HGG tissue at 59.28%. LAT1 was expressed in (D) normal tissue at 59.47%, (E) LGG tissue at 5.17%, and (F) HGG tissue at 17.24%. In the endothelium, ASCT2 was expressed in (A) normal tissue at 0%, (B) LGG tissue at 65.96%, and (C) HGG tissue at 98.2%. LAT1 was expressed in the endothelium of (A) normal tissue at 54.1%, (B) LGG tissue at 69.42%, and (C) HGG tissue at 100%. ASCT2, alanine-serine-cysteine transporter 2; LAT1, L-type amino acid transporter 1; LGG, low-grade glioma; HGG, high-grade glioma

QuPath analysis revealed that ASCT2 was expressed in normal tissue at 2.05%, in LGG tissue at 1.22%, and in HGG tissue at 59.28% (Figure 2). LAT1 was expressed in normal tissue at 59.47%, in LGG tissue at 5.17%, and in HGG tissue at 17.24% (Figure 2). These findings support the classifications designated by the pathologists at HPA.

At the endothelium, ASCT2 was expressed in normal tissue at 0%, in LGG at 65.96%, and in HGG at 98.2% (Figure 2). LAT1 was expressed in the endothelium of normal tissue at 54.1%, in LGG tissue at 69.42%, and in HGG at 100% (Figure 2).

## Discussion

Compared to other amino acid transporters, LAT1 and ASCT2 are expressed at high levels in a variety of human cancers [2]. These transporters are pivotal for the uptake of essential amino acids, glutamine and leucine, respectively, in tumor cells, though their specific role in glioma remains unclear. ASCT2 and LAT1 may contribute to dysregulation of apoptosis by affecting caspase activity [17], or they may promote tumor growth independently [18].

LAT1 transports large neutral amino acids, and LAT1 knockout has been shown to inhibit cancer cell growth [19]. LAT1 expression is associated with poor survival in several solid tumors [12,20]. ASCT2 transports glutamine, the most highly utilized amino acid in cancer cells [21,22]. High ASCT2 expression has been linked to poorer survival in breast, gastric, and ovarian cancers [21,22], though not specifically in glioma.

In this study, data extracted from TCGA did not demonstrate any clear relationship between mRNA expression of LAT1 or ASCT2 and LGG or HGG, or with survival in either glioma subtype. Although we observed a statistically significant survival benefit with low ASCT2 expression in LGG, this trend did not persist in HGG. Furthermore, unlike the findings reported by Faried, we did not observe any difference in survival based on LAT1 expression [14]. Our study was limited by constraints of the public database, which, although offering a broader sample population, lacked sufficient clinical details for more in-depth analysis.

Conversely, our QuPath analysis of IHC slides from the HPA showed increased staining of LAT1 and ASCT2 in the endothelium of both LGG (Figures 2B, 2E) and HGG (Figures 2C, 2F), with staining intensity correlating with glioma grade. The discrepancy between mRNA expression and IHC findings may be explained by localized mRNA expression within neurons, which rely on compartmentalized translation for complex physiological functions and regulatory processes [23].

As noted earlier, LAT1 has been shown to have very high expression at the BBB, suggesting a regulatory role in neuronal growth. Similarly, in nine out of 10 samples of glioma border zones, LAT1 expression was elevated in infiltrating glioma cells, particularly in the perivascular spaces [24]. Our study demonstrated an even greater differential expression of ASCT2 in the perivascular cells of both LGG and HGG, which may indicate a similar role for this transporter. Larger histologic studies are needed to confirm these findings, as our qualitative analysis was limited by the availability of histopathologic specimens in the HPA.

Both ASCT2 and LAT1 have been investigated for cancer diagnostic imaging using radiolabeled amino acid analogues in positron emission tomography (PET) [25]. These targets may improve PET imaging accuracy, as the commonly used tracer 2-^18^F-fluoro-2-deoxy-D-glucose (^18^F-FDG) has a high false-positive rate [25]. ^18^F-Fluciclovine, a radiolabeled synthetic amino acid that targets both ASCT2 and LAT1, is already used for localizing prostate cancer [26] and has been explored for imaging glioblastoma [27]. Likewise, the LAT1-specific PET probe ^^18^F-FIMP has been described as a pan-cancer biomarker with high specificity for multiple malignancies, including lung, breast, and colon cancers [28-31], and was recently studied in a series of seven patients with glioblastoma [32].

ASCT2 and LAT1 have also been explored as therapeutic targets in glioma [33,34]. LAT1 may be leveraged for the delivery of anti-tumor drugs or as a carrier for radiopharmaceuticals [19,35], or directly targeted for inhibition due to its epitope specificity and role in cancer proliferation [36]. Similarly, selective inhibitors of ASCT2 expression and function are currently under development [36,37].

## Conclusions

Though our study was unable to demonstrate any prognostic significance for mRNA expression of LAT1 or ASCT2, the increased endothelial expression of these transporters supports further investigation into their potential as theranostic targets for glioma. Their localized expression around the tumor vasculature suggests functional relevance in glioma proliferation.
